# Repeated Exposition to Mercury (II) Chloride Enhances Susceptibility to *S. schenckii sensu stricto* Infection in Mice

**DOI:** 10.3390/jof4020064

**Published:** 2018-05-25

**Authors:** Alexander Batista-Duharte, Damiana Téllez-Martínez, Juliana Aparecida Jellmayer, Deivys Leandro Portuondo Fuentes, Marisa Campos Polesi, Amanda Martins Baviera, Iracilda Zeppone Carlos

**Affiliations:** Department of Clinical Analysis, School of Pharmaceutical Sciences, São Paulo State University (UNESP), Rodovia Araraquara-Jaú km 1, Araraquara 14800-903, Brazil; damianatellezm@gmail.com (D.T.-M.); japarecida@gmail.com (J.A.J.); deivysma@gmail.com (D.L.P.F.); marisaoo@gmail.com (M.C.P.); amandamarti@gmail.com (A.M.B.)

**Keywords:** mercury (II) chloride, immunotoxicity, *Sporothrix schenckii* infection, sporotrichosis

## Abstract

Sporotrichosis is a subcutaneous mycosis that has re-emerged in several tropical and subtropical regions over the last decades. Growing findings suggest that the interplay of host, pathogen, and environment has a determinant effect on the diversity, local distribution, and virulence of *Sporothrix schenckii sensu lato*, the etiologic agent. Among the environmental factors, we have studied the potential role of repeated exposures to mercury (Hg), a known immunotoxic xenobiotic that is widely used in gold mining regions where sporotrichosis outbreaks are frequently reported. In this study, male Swiss mice received subcutaneous injections of either 300 or 1200 µg/kg of mercury (II) chloride (HgCl_2_) for 14 days, three times a week. A control group was injected with the vehicle Phosphate Buffered Saline (PBS). Treatment with HgCl_2_ impaired several immunologic parameters that are involved in host response to *Sporothrix* infection, such as the production of TNFα, IL-1, and nitric oxide by macrophages, and Th1/Th2/Th17 populations and their respective cytokines. The consequences of these effects on the host resistance to *S. schenckii* infection were subsequently evaluated. Hg-exposed mice exhibited a higher fungal load in the fungal inoculation site associated to systemic dissemination to spleen and liver on 14 days post-infection and a higher production of specific IgG1 and mild reduction of IgG2a. These findings suggest that repeated exposition to Hg enhances susceptibility to *S. schenckii* infection in mice and can be a factor associated to sporotrichosis outbreaks in endemic and highly Hg-polluted areas.

## 1. Introduction

The effect of chemical pollutants as factors involved in the emergence human fungal diseases has not been described. Mercury (Hg) is a well-known toxic heavy metal and one of the most widespread environmental contaminants. Several species of Hg with different physicochemical properties can be found in air, water, and soils. They include elemental or metallic, inorganic (e.g., mercuric chloride), and organic (e.g., methyl- and ethylmercury) forms, which all have different toxic effects and can be converted from one form to another by natural processes [1]. Epidemiological studies evidenced that Hg may lead to toxic effects even at low concentrations, thus suggesting that a larger proportion of the global population is potentially being affected [2]. Mercury salts are used to amalgamate gold, and a significant amount of Hg is released to the environment during artisanal and small-scale gold mining, the world’s largest anthropogenic source of Hg emission [3,4,5]. Globally, millions of workers are employed as artisanal small-scale gold miners. Numerous studies show that mean Hg concentrations in all exposed subgroups are elevated and above threshold limits, often associated to health problems attributable to chronic metallic Hg exposition [4]. Previous studies demonstrated that exposure to low doses of HgCl_2_ causes immunotoxic effects that promote a higher susceptibility to *Ascaris suum* infections [6], malaria [7], leishmaniasis [8], coxsackievirus infection [9], and borreliosis [10] in murine models.

Sporotrichosis is a subcutaneous mycosis, caused by traumatic inoculation of the thermodimorphic fungus *Sporothrix* spp., including *S. brasiliensis*, *S. globosa*, *S. mexicana*, *S. lurie*, and *S. schenckii sensu stricto* [11]. The disease has a worldwide distribution, although it is more frequent in tropical and subtropical regions. Until recently, sporotrichosis was regarded a neglected disease, but in the last decades, an alarming increase in their incidence has been reported in several hiperendemic areas [12,13,14]. Susceptibility to sporotrichosis and clinical manifestations are highly dependent on the state of the host’s immune system and the presence of different virulence factors [15]. Thus, immunocompromised persons are more susceptible to infection and even more severe forms of the disease [16].

Though the cause of the emergence of this disease is not yet known, some evidences suggest that environmental factors can be involved in modification of fungal virulence and in host susceptibility [17,18]. Outbreaks of sporotrichosis have been described in areas of reduced socioeconomic status and with high levels of contamination. Indeed, *S. schenckii* has been isolated in soils contaminated with heavy metals [19,20]. In addition, several sporotrichosis outbreaks among workers of gold mines since early 20th century have been reported [21,22,23,24,25,26,27]. The largest and most well-documented sporotrichosis outbreaks were described in South Africa between 1941 and 1944 and affected more than 3000 gold mine workers [13,28]. Interestingly, artisanal goldmining is widely practiced in several regions of Latin America, Africa, and Southeast Asia, where sporotrichosis is endemic.

Despite these epidemiological antecedents, there are not studies evaluating the effect of Hg exposition on susceptibility to *S. schenckii* infection. Our hypothesis is that repeated administration to inorganic Hg could impair host resistance to *S. schenckii* infection. This effect was evaluated in a murine model of sporotrichosis.

## 2. Materials and Methods

### 2.1. Animals

Male 5–7-week-old specific-pathogen-free Swiss mice obtained from the Animal House at the School of Pharmaceutical Sciences, UNESP (Araraquara, SP, Brazil), were housed and maintained in microisolator cages (three mice per group). All procedures were approved by the Ethics Committee for Animal Use in Research (Protocol CEUA/FCF/CAR 41/2015) (11st September, 2015) in accordance with the National Institutes of Health Animal Care guidelines (NIH Publications No. 8023, revised 1978).

### 2.2. Experimental Models of HgCl_2_ Exposition

Mercury in the form of inorganic mercury (HgCl_2_) was used for the present study. Mice treated with HgCl_2_ were injected for 14 days, three times a week, subcutaneously with either 300 or 1200 µg/kg of HgCl_2_ (Sigma Chemical Co., St Louis, MO, USA) in 200 µL of sterile phosphate-buffered saline (PBS). Control mice were similarly treated with PBS only [8]. Three independent experiments were performed. Mice were euthanized in a CO_2_ chamber and bled by heart puncture. The resulting sera were aliquoted and stored at −20 °C until use.

### 2.3. Peritoneal Macrophages and Splenocytes Preparation

Peritoneal macrophages and total splenocytes were harvested from treated and control mice as described elsewhere [29,30]. Briefly, peritoneal exudate cells (PECs) were obtained from treated and control mice. Briefly, a 3% aqueous solution of sodium thioglycollate (Difco) was inoculated into the peritoneal cavity of the animals. Three days later, the peritoneal exudates were collected, washed with 5 mL of sterile PBS (pH 7.4), and resuspended in RPMI-1640 complete medium containing 0.02 mM β-mercaptoethanol, 100 U/mL penicillin, 100 U/mL streptomycin, 2 mM l-glutamine, and 5% foetal bovine serum (FBS). The cells were then counted in a Neubauer chamber (Boeco, Germany) and adjusted to a concentration appropriate for each test. Non-adherent cells were removed by incubating the suspension in cell culture plates for 1 h at 37 °C in an atmosphere containing 5% CO_2_ (Forma Scientific, Marietta, OH, USA) and then discarding the supernatant. The remaining adherent cells were incubated at 37 °C, 5% CO_2_ for 24 h, as required for each experiment. For preparation of total splenocytes spleens were aseptically removed and passed through a 100 µm cell strainer into a Petri dish containing 2 mL of PBS with the aid of a syringe plunger. For red cell lysis, the resulting suspension was added with 6 mL of a 0.17 M ammonium chloride solution and then incubated on ice for 5 min. The splenocytes were then separated from the supernatant by centrifugation at 300× *g* for 5 min at 4 °C, washed once with 3 mL of RPMI complete medium, and then resuspended in 1 mL of the same medium. Viability and cell concentration was determined by microscopy using the Trypan blue exclusion test and then the splenocytes were adjusted to 5 × 10^6^ cells/mL in RPMI complete medium.

### 2.4. Blood Urea and Creatinine

Plasma concentrations of urea and creatinine were determined in sera using commercial kits (Labtest Diagnostica S.A., Lagoa Santa, MG, Brazil). The following principles were used: creatinine, via chromogen formation with picrate in alkaline medium [31]; and urea, by the urease method [32]. Assays were performed by spectrophotometric system identification in a semiautomated biochemical analyser.

### 2.5. Nitric Oxide (NO) and Cytokine Measurement

Peritoneal macrophages were incubated for 24 h at 37 °C in the presence of *Escherichia coli* O111B lipopolysaccharide (10 µg/mL) or RPMI-1640C alone for the negative control. To measure NO, 50-µL aliquots of the culture supernatants were mixed with 50 µL of Griess reagent (1% *w*/*v* sulfanilamide, 0.1% *w*/*v* naphthylethylenediamine, and 3% H_3_PO_4_) and incubated at room temperature for 10 min. The color reactions were analyzed at 540 nm with a Multiskan Ascent ELISA reader (Labsystems, Helsinki, Finland). Ex vivo release of macrophage-derived cytokines IL-1β and TNF-α were measured by ELISA (eBioscience, Thermo Fisher Scientific, Waltham, MA, USA) according to the manufacturer’s instructions.

### 2.6. Th1/Th17 Cell Phenotyping and Flow Cytometry Analysis

For surface staining alone, splenocytes were resuspended at a concentration of 1 × 10^6^ cells/mL in staining buffer (PBS 1x and 1% FBS). Fc receptors were blocked by the addition of unlabeled anti-CD16/32 (Fc block; BD Pharmingen, San Diego, CA, USA). The leukocytes were then stained for 20 min at 4 °C with the optimal dilution of FITC anti-mouse CD3 and APC anti-mouse CD4 antibody (BD, Pharmingen). Cells were washed twice with staining buffer, resuspended in 100 µL, and an equal volume of 2% formalin was added to fix the cells. After that, cells were treated with permeabilization buffer and intracellular IFNγ and IL-7A cytokines were identified with PE anti-mouse IL-17A and PE-Cy7- anti-mouse IFNγ respectively (BD, Pharmingen). The stained cells were analysed with a BDAccuri C6 flow cytometer (BD Biosciences, San Jose, CA, USA).

### 2.7. Th1/Th2/Th17-Related Cytokines Analysis by Cytometric Bead Array (CBA)

Cytokines in supernatant of splenocytes stimulated with Concanavalin A (ConA) (0.25 µg/mL) or unstimulated were measured with BD CBA Mouse Th1/Th2/Th17 Cytokine Kit (BD Bioscience, San Jose, CA, USA). The kit was used for the simultaneous detection of mouse interleukin-2 (IL-2), interleukin-4 (IL-4), interleukin-6 (IL-6), interferon-γ (IFN-γ), tumor necrosis factor (TNF), interleukin-17A (IL-17A), and interleukin-10 (IL-10) in a single sample. The operations were performed according to the manufacturer’s instruction and the analysis was made with a BDAccuri C6 flow cytometer (BD Biosciences).

### 2.8. Microorganism and Growth Conditions

*S. schenckii* strain 1099–18 (ATCC MYA-4821), kindly provided by the Oswaldo Cruz Foundation, Rio de Janeiro, Brazil. For in vivo experiments, the mycelia phase of each strain was grown in Sabouraud broth (Difco, Detroit, MI, USA) for 5 days at room temperature, and the conidia were isolated from hyphae using a Buchner funnel and sterile gauze [33].

### 2.9. Experimental Infection

Three mice from treated groups and one untreated group were subcutaneously inoculated in the dorsal sacral region with 1 × 10^7^ conidia suspended in 200 µL of PBS. A non-infected group inoculated with PBS was included. The parameters determined to evaluate the severity of the infection were the fungal load in the primary skin lesion and the systemic dissemination in spleen and liver by colony forming unit count (CFU) in Mycosel Agar after euthanasia 14th day post-infection [33]. Three independent experiments were performed.

### 2.10. Quantification of IgG anti Cell Wall Proteins of S. schenckii (ssCWP) by ELISA

Quantification of ssCWP-specific IgG in serum from mice treated with HgCl_2_ and infected, was carried out by ELISA as described by Portuondo et al. (2016) [34]. Sera from non-treated and non-infected mice control were used as negative control.

### 2.11. Statistical Analysis

Data were analysed using one-way analysis of variance (ANOVA) followed by Tukey’s post-test using Graph Pad Prism 5 (GraphPad Software Inc., San Diego, CA, USA). *p* < 0.05 was considered statistically significant.

## 3. Results

### 3.1. General Toxicity Parameters

**Figure 1:**
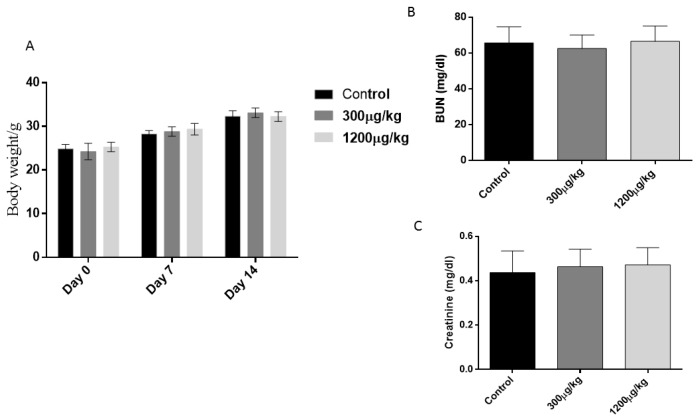
Average absolute body weight (g) per week across the duration of the study (**A**), and Serum total urea and creatinine concentration (**B**,**C**) in Swiss mice treated with either 300 or 1200 µg/kg of HgCl_2_, subcutaneously for 14 days, three times a week. Data expressed as means plus standard errors. No differences were observed between groups (*p* > 0.05).

Treated animals did not exhibit weight loss or decreased food or water intake, which are symptoms of mercury intoxication. In addition, there was no alteration of urea and creatinine concentration in sera (*p* > 0.05) as evidence of the absence of renal lesion (Figure 1).

### 3.2. Mercury Reduced NO and IL-1 While Stimulated TNFα Production by Macrophages

**Figure 2:**
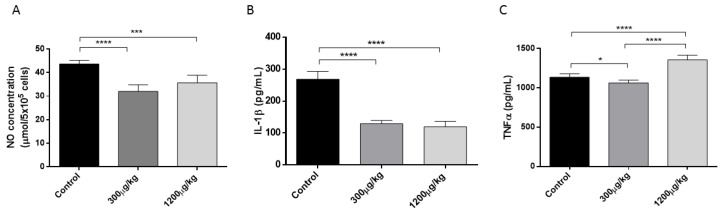
NO (**A**); IL-1β (**B**) and TNFα (**C**) production in cultured peritoneal macrophages from Swiss mice treated with either PBS (Control), 300 or 1200 µg/kg of HgCl_2_, subcutaneously for 14 days, three times a week. Peritoneal exudate cells (PECs) were stimulated overnight with LPS. The data are shown as the mean ± SD from three independent experiments (* *p* < 0.05; *** *p* < 0.001; **** *p* < 0.0001).

Peritoneal macrophages from treated mice with HgCl_2_ exhibited a dose-dependent reduction of NO, and IL-1 production, associated to higher TNFα release when compared with non-treated mice (*p* < 0.05) (Figure 2).

### 3.3. Mercury Caused Reduction of CD3+CD4+, Th1 and Th17 Lymphocytes

**Figure 3:**
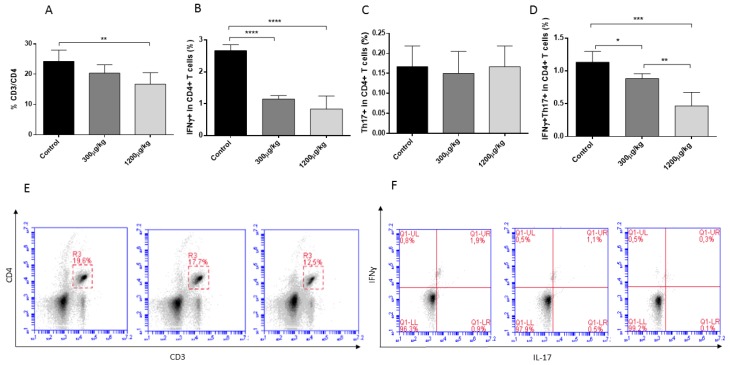
Quantification of CD3+CD4+ lymphocytes (**A**) and Th1 (**B**), Th17 (**C**), Th1/Th17 (**D**) subpopulations by intracellular staining of IFNγ+ and IL-17A+ in splenocytes from male Swiss mice treated with either PBS (Control), 300 or 1200 µg/kg of HgCl_2_, subcutaneously for 14 days, three times a week (* *p* < 0.05, ** *p* < 0.01; *** *p* < 0.001; **** *p* < 0.0001). E and F depict representative plots and gating strategy for determination of CD3+CD4+ (**E**), IFNγ+, IL-17A+, or IFNγ+IL-17+ Th cells (**F**).

In this study, a detriment effect of Hg treatment against CD3+CD4+, Th1, and Th1/Th17 lymphocytes was detected in the treated groups. The analysis of IFNγ+, IL-17A+, and IFNγ+IL-17A+ cells from CD3+CD4+ lymphocytes revealed a significant reduction of IFNγ+ lymphocytes in both treated group of mice and a dose-dependent reduction of CD3+CD4+ lymphocytes and IFNγ+IL-17A+ population (*p* < 0.05) (Figure 3).

### 3.4. Mercury Inhibited the Splenocytes Proliferation and Reduced Th1/Th2/Th17 Cytokine Production

**Figure 4:**
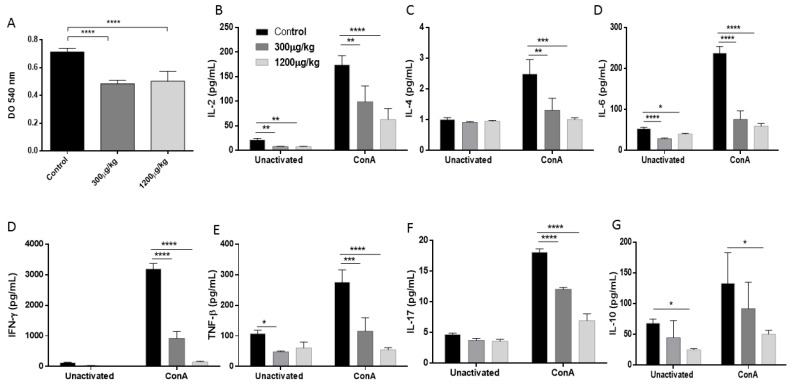
Proliferative response (**A**) and ex vivo release of Th1/Th2/Th17 cytokines production (**B**–**G**), of splenocytes unstimulated or after ConA. Splenocytes were purified from the spleen of male Swiss mice treated with either PBS (Control), 300 or 1200 µg/kg of HgCl_2_, subcutaneously for 14 days, three times a week (* *p* < 0.05; ** *p* < 0.01; *** *p* < 0.001; **** *p* < 0.0001).

Because Hg promoted a reduction of Th1 and Th1/Th17 populations, we investigated whether the production of their respective cytokines and those produced by Th2 lymphocytes are also affected. A dose-dependent reduction in the production of IFNγ and TNF-β (Th1 profile); IL-2, IL-4, IL-6 and IL-10 (Th2 profile), and IL-17 (Th17 profile) was observed in the culture supernatant of splenocytes from mice treated with Hg, suggesting a reduced activity of the Th1/Th2 Th17 response (*p* < 0.05). This effect was associated to a drop of splenocytes proliferative activity after ex vivo polyclonal stimulation with ConA (Figure 4).

### 3.5. Mercury Enhanced Susceptibility to S. schenckii Infection

To determine whether immunotoxic effects promoted by repeated administration of Hg could alter host responses to *S. schenckii*, groups of mice received injections of either PBS or HgCl_2_ as previously described, and after two weeks of treatment, mice were infected using the natural way of infection [33]. After subcutaneous inoculation of 10^7^ conidia, the progression of the disease was followed up to 14 days post-infection. The first clinical manifestations of local infection began to be observed from the third day of inoculation. For a better observation of the local changes the mice were depilated before the inoculation of the fungus. The fungal burden in the primary subcutaneous lesion was determined by CFU. Additionally, possible dissemination to the internal organs (spleen and liver) was investigated.

**Figure 5:**
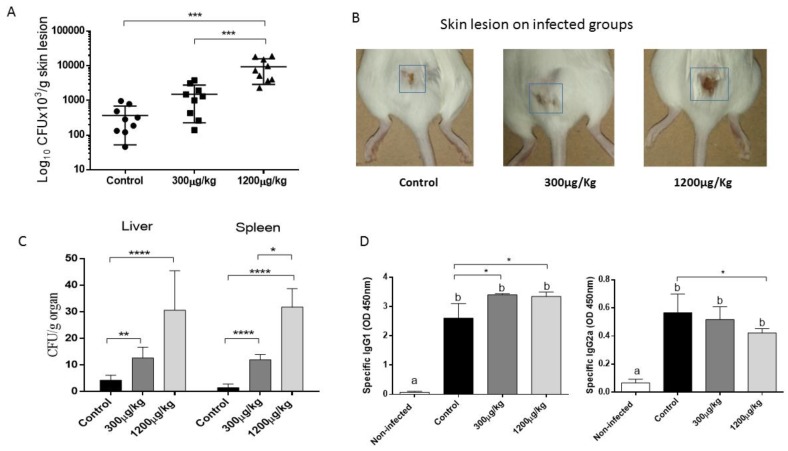
Fungal load in the inoculation site (**A**). Representative images of external aspect of skin lesion from each experimental group (**B**), fungal load in spleen and liver (**C**), IgG1 and IgG2a level in sera (**D**) of male Swiss mice treated with either PBS (Control), 300 or 1200 µg/kg of HgCl_2_, subcutaneously for 14 days, three times a week. The analysis was made on 14 days post fungal inoculation (* *p* < 0.05; ** *p* < 0.01; *** *p* < 0.001; **** *p* < 0.0001). Different letters above the bars represent significant differences (*p* < 0.05) between non-infected and infected groups.

Mice treated with the highest dose of HgCl_2_ showed a greater ulcerative lesion on the skin, associated to higher fungal load in the inoculation site while. All treated mice developed systemic spread, some of them exhibited a high systemic dissemination to spleen and liver at 14 days post-infection in the highest dose of Hg. The animals that did not receive Hg treatment showed little spread to the liver and spleen. In addition, a higher production of specific IgG1 and IgG2a was observed in the treated groups without differences between them, in comparison with non-treated animals (*p* < 0.05) (Figure 5).

## 4. Discussion

The exposition to Hg, even in small amounts, can have harmful effects in different systems including the immune systems [2]. Individuals living in Hg-polluted areas can have daily Hg exposition which easily exceed the WHO recommended limit of 0–43 mg/kg body weight and may be as high as 100 mg a day [8]. The immunotoxic hazards of Hg compounds have been extensively demonstrated in animal models and epidemiological studies, and Hg appears to have the most diverse effects on the immune system including autoimmune dysfunction and immunosuppression [35,36,37]. However, understanding the risks associated with Hg exposure is complicated by the existence of several Hg species in the environment, and the overarching influences of environmental, biological, and socioeconomic factors [38].

Several experimental studies have evidenced that repeated administration of inorganic Hg enhance the susceptibility to several infections and parasitic diseases [6,7,8,9,10]. The high frequency of sporotrichosis outbreaks among gold mine workers and the lack of information about the effect of Hg exposition on susceptibility to this disease, prompted us to study the influence of inorganic Hg, an immunotoxic metal on the host resistance to *S. schenckii* infection. The selected experimental model of subcutaneous inoculation of HgCl_2,_ represent a proof of concept that have been used during decades to measure the toxic effect of Hg and the host susceptibility to infections after exposition [39,40,41,42,43]. The doses of HgCl_2_ used in this study were selected as a minimum regime reported to alter immune function in mice without relevant toxicity manifestation in other systems [7,8,44].

Firstly, we explored the effect of repeated exposition of two doses Hg on several known mechanisms involved in anti-*S. schenckii* immune response. Macrophages are involved in the early clearance of *S. schenckii* during infection. They produce a variety of effector molecules, including TNF-α, IL-1, IL-6, and NO that participate in fungal elimination [45,46,47]. In this study, peritoneal macrophages from mice treated with Hg exhibited reduced capacity of NO and IL-1 production but enhanced TNFα production. An inhibitory effect of Hg on NO production associated to stimulation of TNFα pathway was reported in the murine macrophage cell line J774A.1 treated in vitro with HgCl_2_ in presence or absence of LPS. The authors demonstrated that Hg directly activated p38MAPK signalling with increased LPS-induced p38MAPK activation as a possible mechanism of TNFα stimulation [48]. More recently, it was reported that administration of methylmercury, another environmental chemical species of Hg, selectively induced TNF-α expression in the brain of mice [49]. Another study showed that inorganic Hg caused upregulation of TNFα, IL-10, and TGF-β mRNAs and downregulation of IL-1β in the head kidney of yellow catfish following 6 weeks of exposure to environmental concentrations of the metal [50]. In reference to the NO inhibition, authors suggested that the thiol-binding properties of Hg may be responsible for the inhibitory effects on NF-κB activation and downstream NO production [48,51,52]. However, a study performed in RAW264.7 cells (a murine monocytic cell line) revealed a contrary effect; Hg induced the activation of NF-κB and the expression of iNOS and COX-2 [53]. Therefore, whether or not mercury inhibits NF-κB activation is controversial and further studies are needed for understanding this effect.

A significant reduction of CD3+CD4+ lymphocytes and Th1/Th7 subpopulations was also observed in mice exposed to Hg. The treatment with Hg also affected the production of Th1/Th2/Th17 after splenocytes stimulation with ConA. All these finding are consistent with specific immunotoxic effects of mercury related to depressed cell mediated immunity [7,37]. However, several authors reported that Hg treatment favours a Th2 response in certain mice models [7,8,54], but in the conditions used in this study with the Swiss mice, this effect was not observed.

Different studies showed that the function of Th1 and Th17 lymphocytes are determinant for *S. schenckii* clearance during the infection process [30,55,56,57]. In this way, treatment with anti-IL-23 mAb caused a marked decrease in IL-17, IL-22, and IFNγ, which was shown to be directly correlated with an impaired capacity to control the *S. schenckii* infection, in a murine model [30]. Similarly, in a model of immunosuppression by cyclophosphamide with damage in diverse populations of T lymphocytes and reduced production capacity of IFN and IL-17, a significant increase in susceptibility to subcutaneous infection by *S. schenckii* was observed [58].

Based on these results we hypothesized that impaired immunity caused by HgCl_2_ exposure can enhance susceptibility to *S. schenckii* infection. The hypothesis was confirmed when treated and non-treated groups were infected with *S. schenckii* conidia by subcutaneous route, the natural way of infection [33]. The higher fungal load in the inoculation site associated to and stronger systemic dissemination on 14 days post-infection on treated groups suggest that the deleterious effects of Hg in the anti-*Sporothrix* mechanisms reduced the resistance to the infection in our model. On the other hand, a higher production of specific IgG1 associated to a mild reduction of IgG2a in Hg-exposed mice was also observed. Studies in our laboratory have shown that IgG1 and IgG2a antibodies against *S. schenckii* play a role against fungal infection [34,59]. However, the elevated production of specific IgG1 observed in this study was not sufficient to achieve an effective control of the infection.

Several reports reveal that repeated exposure to Hg is capable of raising IgG1 values and this elevation has been associated with an increase in the production of IL-4 and IgE as well as autoimmune manifestations [8,60,61]. However, in our study, a reduction of IL-4 was detected in the supernatant of splenocytes from mice treated with Hg and stimulated with the T cell mitogen, ConA, albeit de Vos et al. (2007) reporting that Con A had relatively little effect on IL-4 production in vitro [62].

On the other hand, the slight reduction of IgG2a can be associated to the observed reduction of Th1 response in mice that were treated with HgCl_2_, since Th1 response is associated with the induction of IgG2a, IgG2b, and IgG3 antibodies [63]. It is known that the patterns of response to treatment with Hg are complex and highly dependent of the genetic pattern and the susceptibility of the animals used [64]. For this reason, in this study the outbreed Swiss mice was used. These mice exhibit a diverse genetic background that mimics the high degree of heterozygosity found in typical human populations.

In this first study, we wanted to have an approach to the possible effect of the administration of Hg in immunotoxic dose on susceptibility to infection by *S. schenckii*. However, in practice, many populations exposed to this metal can exhibit different levels of exposure and frequently the Hg levels may be above the values used in this experiment. Gibb et al. (2014) reviewed more than 60 studies that measured biomarkers of Hg exposure in individuals living in or near artisanal small-scale gold mining communities in 19 different countries in South America, Asia, and Africa. These studies demonstrated that hair and urine concentrations of Hg are well above World Health Organization health guidance values for these communities [65].

In addition to the high incidence of sporotrichosis here discussed, other frequent infections have been reported in gold miners, including tuberculosis, viral infections, and malaria [66,67]. The multiple alterations that Hg causes in the immune system and that have also been described in these populations [68] may be favouring the occurrence of different types of opportunistic infections.

In conclusion, the results of the present study suggest that exposures to repeated doses of Hg can compromise host immune response to *S. schenckii* causing enhanced susceptibility to sporotrichosis in mice used in this study. This effect can be caused, at least in part, by downregulation of macrophages function and inhibition of antifungal Th1 and Th17 responses. Further studies with other Hg species are necessary, associating exposure, Hg levels in blood and other tissues, and studies of different immunological endpoints and fungal load in animals and humans. These studies will allow a better definition of risk related to Hg exposition in contaminated areas and sporotrichosis including their different clinical forms.

## References

[1] Gai K., Hoelen T.P., Hsu-Kim H., Lowry G.V. (2016). Mobility of Four Common Mercury Species in Model and Natural Unsaturated Soils. Environ. Sci. Technol..

[2] World Health Organization (WHO) (2007). Exposure to Mercury: A Major Public Health Concern WHO, Public Health and Environment.

[3] Kristensen A.K., Thomsen J.F., Mikkelsen S. (2014). A review of mercury exposure among artisanal small-scale gold miners in developing countries. Int. Arch. Occup. Environ. Health.

[4] Steckling N., Tobollik M., Plass D., Hornberg C., Ericson B., Fuller R., Bose-O’Reilly S. (2017). Global Burden of Disease of Mercury Used in Artisanal Small-Scale Gold Mining. Ann. Glob. Health.

[5] Esdaile L.J., Chalker J.M. (2018). The Mercury Problem in Artisanal and Small-Scale Gold Mining. Chemistry.

[6] Borosková Z., Soltys J., Benková M. (1995). Effect of mercury on the immune response and mean intensity of Ascaris suum infection in guinea pigs. J. Helminthol..

[7] Silbergeld E.K., Sacci J.B., Azad A.F. (2000). Mercury exposure and murine response to Plasmodium yoelii infection and immunization. Immunopharmacol. Immunotoxicol..

[8] Bagenstose L.M., Mentink-Kane M.M., Brittingham A., Mosser D.M., Monestier M. (2001). Mercury enhances susceptibility to murine leishmaniasis. Parasite Immunol..

[9] South P.K., Morris V.C., Levander O.A., Smith A.D. (2001). Mortality in mice infected with an amyocarditic coxsackievirus and given a subacute dose of mercuric chloride. J. Toxicol. Environ. Health A.

[10] Ekerfelt C., Andersson M., Olausson A., Bergström S., Hultman P. (2007). Mercury exposure as a model for deviation of cytokine responses in experimental Lyme arthritis: HgCl_2_ treatment decreases T helper cell type 1-like responses and arthritis severity but delays eradication of Borrelia burgdorferi in C3H/HeN mice. Clin. Exp. Immunol..

[11] Orofino-Costa R., Macedo P.M., Rodrigues A.M., Bernardes-Engemann A.R. (2017). Sporotrichosis: An update on epidemiology, etiopathogenesis, laboratory and clinical therapeutics. An. Bras. Dermatol..

[12] Carlos I.Z., Batista-Duharte A. (2015). Sporotrichosis: An emergent disease. Sporotrichosis.

[13] Chakrabarti A., Bonifaz A., Gutierrez-Galhardo M.C., Mochizuki T., Li S. (2015). Global epidemiology of sporotrichosis. Med. Mycol..

[14] Lopes-Bezerra L.M., Mora-Montes H.M., Zhang Y., Nino-Vega G., Rodrigues A.M., de Camargo Z.P., de Hoog S. (2018). Sporotrichosis between 1898 and 2017: The evolution of knowledge on a changeable disease and on emerging etiological agents. Med. Mycol..

[15] Almeida-Paes R., de Oliveira L.C., Oliveira M.M., Gutierrez-Galhardo M.C., Nosanchuk J.D., Zancopé-Oliveira R.M. (2015). Phenotypic characteristics associated with virulence of clinical isolates from the Sporothrix complex. Biomed. Res. Int..

[16] Lee P.P., Lau Y.L. (2017). Cellular and Molecular Defects Underlying Invasive Fungal Infections-Revelations from Endemic Mycoses. Front. Immunol..

[17] Batista-Duharte A., Martínez D.T., da Graça Sgarbi D.B., Carlos I.Z., Zeppone Carlos I. (2015). Environmental Conditions and Fungal Pathogenicity. Sporotrichosis.

[18] Téllez M.D., Batista-Duharte A., Portuondo D., Quinello C., Bonne-Hernández R., Carlos I.Z. (2014). *Sporothrix schenckii* complex biology: Environment and fungal pathogenicity. Microbiology.

[19] Dixon D.M., Salkin I.F., Duncan R.A., Hurd N.J., Haines J.H., Kemna M.E., Coles F.B. (1991). Isolation and characterization of *Sporothrix schenckii* from clinical and environmental sources associated with the largest U.S. epidemic of sporotrichosis. J. Clin. Microbiol..

[20] Ulfig K. (1994). The occurrence of keratinolytic fungi in the polluted environment of the Labedy district in Gliwice. Rocz. Panstw. Zakl. Hig..

[21] Pijper A., Pulinger D.B. (1927). An outbreak of sporotrichosis among South African native miners. Lancet.

[22] Dangerfield L.F., Gear J. (1941). Sporotrichosis among miners on the Witwatersrand gold mines. S. Afr. Med. J..

[23] Findlay G.H. (1985). Sporotrichosis research in the Transvaal—How it began 60 years ago. S. Afr. Med. J..

[24] Findlay G.H. (1970). The epidemiology of sporotrichosis in the Transvaal. Sabouraudia.

[25] Helm M.A.F., Berman C. (1947). The clinical, therapeutic and epidemiological features of the sporotrichosis infection on the mines. Sporotrichosis Infection on Mines of the Witwatersrand.

[26] Quintal D. (2000). Sporotrichosis infection on mines of the Witwatersrand. J. Cutan. Med. Surg..

[27] Govender N.P., Maphanga T.G., Zulu T.G., Patel J., Walaza S., Jacobs C., Ebonwu J.I., Ntuli S., Naicker S.D., Thomas J. (2015). An outbreak of lymphocutaneous sporotrichosis among mine-workers in South Africa. PLoS Negl. Trop. Dis..

[28] Queiroz-Telles F., McGinnis M.R., Salkin I., Graybill J.R. (2003). Subcutaneous mycoses. Infect. Dis. Clin. N. Am..

[29] Gonçalves A.C., Maia D.C., Ferreira L.S., Monnazzi L.G., Alegranci P., Placeres M.C., Batista-Duharte A., Carlos I.Z. (2015). Involvement of major components from *Sporothrix schenckii* cell wall in the caspase-1 activation, nitric oxide and cytokines production during experimental sporotrichosis. Mycopathologia.

[30] Ferreira L.S., Gonçalves A.C., Portuondo D.L., Maia D.C., Placeres M.C., Batista-Duharte A., Carlos I.Z. (2015). Optimal clearance of *Sporothrix schenckii* requires an intact Th17 response in a mouse model of systemic infection. Immunobiology.

[31] Cook J.G.H. (1971). Creatinine assay in the presence of protein. Clin. Chim. Acta.

[32] Bernt E., Bergmeyer H.U., Bergmeyer H.U. (1965). Urea, p 401–406. Methods of Enzymatic Analysis.

[33] Castro R.A., Kubitschek-Barreira P.H., Teixeira P.A., Sanches G.F., Teixeira M.M., Quintella L.P., Almeida S.R., Costa R.O., Camargo Z.P., Felipe M.S. (2013). Differences in cell morphometry, cell wall topography and Gp70 expression correlate with the virulence of Sporothrix brasiliensis clinical isolates. PLoS ONE.

[34] Portuondo D.L., Batista-Duharte A., Ferreira L.S., Martínez D.T., Polesi M.C., Duarte R.A., de Paula E., Silva A.C., Marcos C.M., Almeida A.M. (2016). A cell wall protein-based vaccine candidate induce protective immune response against *Sporothrix schenckii* infection. Immunobiology.

[35] Silbergeld E.K., Silva I.A., Nyland J.F. (2005). Mercury and autoimmunity: Implications for occupational and environmental health. Toxicol. Appl. Pharmacol..

[36] Silva I.A., Nyland J.F., Gorman A., Perisse A., Ventura A.M., Santos E.C., Souza J.M., Burek C.L., Rose N.R., Silbergeld E.K. (2004). Mercury exposure, malaria, and serum antinuclear/antinucleolar antibodies in Amazon populations in Brazil: A cross-sectional study. Environ. Health.

[37] Silva I.A., Graber J., Nyland J.F., Silbergeld E.K. (2005). In vitro HgCl_2_ exposure of immune cells at different stages of maturation: Effects on phenotype and function. Environ. Res..

[38] Eagles-Smith C.A., Silbergeld E.K., Basu N., Bustamante P., Diaz-Barriga F., Hopkins W.A., Kidd K.A., Nyland J.F. (2018). Modulators of mercury risk to wildlife and humans in the context of rapid global change. Ambio.

[39] Gray J.A., Kavlock R.J. (1987). Pharmacologic probing of mercuric chloride-induced renal dysfunction in the neonatal rat. J. Pharmacol. Exp. Ther..

[40] Hultman P., Eneström S. (1988). Mercury induced antinuclear antibodies in mice: Characterization and correlation with renal immune complex deposits. Clin. Exp. Immunol..

[41] Hultman P., Bell L.J., Eneström S., Pollard K.M. (1993). Murine susceptibility to mercury. II. autoantibody profiles and renal immune deposits in hybrid, backcross, and H-2d congenic mice. Clin. Immunol. Immunopathol..

[42] Fiuza Tda L., Oliveira C.S., da Costa M., Oliveira V.A., Zeni G., Pereira M.E. (2015). Effectiveness of (PhSe)2 in protect against the HgCl_2_ toxicity. J. Trace Elem. Med. Biol..

[43] Crowe W., Allsopp P.J., Watson G.E., Magee P.J., Strain J.J., Armstrong D.J., Ball E., McSorley E.M. (2017). Mercury as an environmental stimulus in the development of autoimmunity—A systematic review. Autoimmun. Rev..

[44] Dieter M., Luster M.I., Boorman G.A. (1983). Immunological and biochemical responses in mice treated with mercuric chloride. Toxicol. Appl. Pharmacol..

[45] Carlos I.Z., Zini M.M., Sgarbi D.B., Angluster J., Alviano C.S., Silva C.L. (1994). Disturbances in the production of interleukin-1 and tumor necrosis factor in disseminated murine sporotrichosis. Mycopathologia.

[46] Maia D.C., Gonçalves A.C., Ferreira L.S., Manente F.A., Portuondo D.L., Vellosa J.C., Polesi M.C., Batista-Duharte A., Carlos I.Z. (2016). Response of Cytokines and Hydrogen Peroxide to *Sporothrix schenckii* Exoantigen in Systemic Experimental Infection. Mycopathologia.

[47] Jellmayer J.A., Ferreira L.S., Manente F.A., Gonçalves A.C., Polesi M.C., Batista-Duharte A., Carlos I.Z. (2017). Dectin-1 expression by macrophages and related antifungal mechanisms in a murine model of *Sporothrix schenckii* sensu stricto systemic infection. Microb. Pathog..

[48] Kim S.H., Johnson V.J., Sharma R.P. (2002). Mercury inhibits nitric oxide production but activates proinflammatory cytokine expression in murine macrophage: Differential modulation of NF-kappaB and p38 MAPK signaling pathways. Nitric Oxide.

[49] Iwai-Shimada M., Takahashi T., Kim M.S., Fujimura M., Ito H., Toyama T., Naganuma A., Hwang G.W. (2016). Methylmercury induces the expression of TNF-α selectively in the brain of mice. Sci. Rep..

[50] Sun Y., Li Y., Rao J., Liu Z., Chen Q. (2018). Effects of inorganic mercury exposure on histological structure, antioxidant status and immune response of immune organs in yellow catfish (*Pelteobagrus fulvidraco*). J. Appl. Toxicol..

[51] Dieguez-Acuña F.J., Woods J.S. (2000). Inhibition of NF-κB-DNA binding by mercuric ion: Utility of the non-thiol reductant, tris(2-carboxyethyl)phosphine hydrochloride (TCEP), on detection of impaired NF-kappaB-DNA binding by thiol-directed agents. Toxicol. In Vitro.

[52] Shumilla J.A., Wetterhahn K.E., Barchowsky A. (1998). Inhibition of NF-κB binding to DNA by chromium, cadmium, mercury, zinc, and arsenite in vitro: Evidence of a thiol mechanism. Arch. Biochem. Biophys..

[53] Park H.J., Youn H.S. (2013). Mercury induces the expression of cyclooxygenase-2 and inducible nitric oxide synthase. Toxicol. Ind. Health.

[54] Fournié G.J., Saoudi A., Druet P., Pelletier L. (2002). Th2-type immunopathological manifestations induced by mercury chloride or gold salts in the rat: Signal transduction pathways, cellular mechanisms and genetic control. Autoimmun. Rev..

[55] Maia D.C.G., Sassá M.F., Placeres M.C.P., Carlos I.Z. (2006). Influence of Th1/Th2 cytokines and nitric oxide in murine systemic infection induced by *Sporothrix schenckii*. Mycopathologia.

[56] Uenotsuchi T., Takeuchi S., Matsuda T., Urabe K., Koga T., Uchi H., Nakahara T., Fukagawa S., Kawasaki M., Kajiwara H. (2006). Differential induction of Th1-prone immunity by human dendritic cells activated with *Sporothrix schenckii* of cutaneous and visceral origins to determine their different virulence. Int. Immunol..

[57] Flores-García A., Velarde-Félix J.S., Garibaldi-Becerra V., Rangel-Villalobos H., Torres-Bugarín O., Zepeda-Carrillo E.A., Ruíz-Bernés S., Ochoa-Ramírez L.A. (2015). Recombinant murine IL-12 promotes a protective Th1/cellular response in Mongolian gerbils infected with *Sporothrix schenckii*. J. Chemother..

[58] Manente F.A., Quinello C., Ferreira L.S., de Andrade C.R., Jellmayer J.A., Portuondo D.L., Batista-Duharte A., Carlos I.Z. (2017). Experimental sporotrichosis in a cyclophosphamide-induced immunosuppressed mice model. Med. Mycol..

[59] Portuondo D.L., Batista-Duharte A., Ferreira L.S., de Andrade C.R., Quinello C., Téllez-Martínez D., de Aguiar Loesch M.L., Carlos I.Z. (2017). Comparative efficacy and toxicity of two vaccine candidates against *Sporothrix schenckii* using either Montanide™ Pet Gel A or aluminum hydroxide adjuvants in mice. Vaccine.

[60] Ochel M., Vohr H.W., Pfeiffer C., Gleichmann E. (1991). IL-4 is required for the IgE and IgG1 increase and IgG1 autoantibody formation in mice treated with mercuric chloride. J. Immunol..

[61] Arefieva A.S., Kamaeva A.G., Krasilshchikova M.S. (2016). Low doses of mercuric chloride cause the main features of anti-nucleolar autoimmunity in female outbred CFW mice. Toxicol. Ind. Health.

[62] De Vos G., Abotaga S., Liao Z., Jerschow E., Rosenstreich D. (2007). Selective effect of mercury on Th2-type cytokine production in humans. Immunopharmacol. Immunotoxicol..

[63] Germann T., Bongartz M., Dlugonska H., Hess H., Schmitt E., Kolbe L., Kölsch E., Podlaski F.J., Gately M.K., Rüde E. (1995). Interleukin-12 profoundly up-regulates the synthesis of antigen-specific complement-fixing IgG2a, IgG2b and IgG3 antibody subclasses in vivo. Eur. J. Immunol..

[64] Abedi-Valugerdi M., Möller G. (2000). Contribution of H-2 and non-H-2 genes in the control of mercury-induced autoimmunity. Int. Immunol..

[65] Gibb H., O’Leary K.G. (2014). Mercury exposure and health impacts among individuals in the artisanal and small-scale gold mining community: A comprehensive review. Environ. Health Perspect..

[66] Eisler R. (2003). Health risks of gold miners: A synoptic review. Environ. Geochem. Health.

[67] Douine M., Mosnier E., Le Hingrat Q., Charpentier C., Corlin F., Hureau L., Adenis A., Lazrek Y., Niemetsky F., Aucouturier A.L. (2017). Illegal gold miners in French Guiana: A neglected population with poor health. BMC Public Health.

[68] Lubick N. (2010). Mercury alters immune system response in artisanal gold miners. Environ. Health Perspect..

